# A Glance at Recombination Hotspots in the Domestic Cat

**DOI:** 10.1371/journal.pone.0148710

**Published:** 2016-02-09

**Authors:** Hasan Alhaddad, Chi Zhang, Bruce Rannala, Leslie A. Lyons

**Affiliations:** 1 College of Science, Department of Biological Sciences, Kuwait University, Safat, 13060, Kuwait; 2 Department of Bioinformatics and Genetics, Swedish Museum of Natural History, Box 50007, SE-104 05, Stockholm, Sweden; 3 Department of Evolution and Ecology, University of California Davis, Davis, CA, 95616, United States of America; 4 Department of Veterinary Medicine and Surgery, College of Veterinary Medicine, University of Missouri-Columbia, Columbia, MO, 65211, United States of America; Texas A&M University, UNITED STATES

## Abstract

Recombination has essential roles in increasing genetic variability within a population and in ensuring successful meiotic events. The objective of this study is to (i) infer the population-scaled recombination rate (*ρ*), and (ii) identify and characterize regions of increased recombination rate for the domestic cat, *Felis silvestris catus*. SNPs (*n* = 701) were genotyped in twenty-two East Asian feral cats (random bred). The SNPs covered ten different chromosomal regions (A1, A2, B3, C2, D1, D2, D4, E2, F2, X) with an average region size of 850 Kb and an average SNP density of 70 SNPs/region. The Bayesian method in the program *inferRho* was used to infer regional population recombination rates and hotspots localities. The regions exhibited variable population recombination rates and four decisive recombination hotspots were identified on cat chromosome A2, D1, and E2 regions. As a description of the identified hotspots, no correlation was detected between the GC content and the locality of recombination spots, and the hotspots enclosed L2 LINE elements and MIR and tRNA-Lys SINE elements.

## Introduction

Recombination is a major source of genetic variation within sexually reproducing organisms, and is necessary for the proper alignment and segregation of homologous chromosomes during meiosis. New combinations of parental alleles across loci are generated via recombination and transmitted to succeeding generations. Lack of recombination may cause failure of meiotic division, or formation of gametes with chromosome number abnormalities (aneuploidy), which are often detrimental.

Localized chromosomal regions with high recombination rates relative to surrounding areas are referred to as recombination “hotspots” [1]. Known hotspots in mice and human are generally 1–2 kb regions of high recombination rates surrounded by regions of low recombination [2]. In humans, recombination hotspots are distributed about every 200 Kb [3] and over 25 000 hotspots have been identified [4]. The first human recombination hotspot was identified using restriction site polymorphisms in β-globin gene cluster [5]. A higher resolution human hotspot was localized using sperm typing via PCR in a region that harbors GC-rich mini-satellite (MS32) as a molecular signature [6].

Several genomic features exhibit correlations with recombination hotspots. The GC content has been found to be positively correlated with recombination hotspots in humans [4], dog [7], pig [8], and chicken [9] but not in mice [10]. Long terminal repeats (LTR), long interspersed elements (LINE), and short interspersed elements (SINE) were also observed to be positively correlated with the locality of recombination hot spots in human [4,11], mice [10] and pigs [8]. DNA motifs (*cis*) have been identified to be associated with recombination hot spots both in humans [4,12,13] and in other organisms [14]. In addition to the *cis* elements presented by the motifs, a *trans* element, PRDM9, was found to be a major determinant of recombination hotspots in human and mice [15,16] but not in dog and related wild relatives [7,17,18]. PRDM9 is thought to bind to a DNA motif via a zinc finger domain, altering the chromatin structure through methylation, and recruiting recombination molecular machinery [19,20].

Coarse-scale recombination in cats has previously been investigated through linkage map analyses using sparsely distributed microsatellite markers [21,22]. The objective of this study is to investigate fine-scale recombination rates, and recombination hotspots, in the domestic cat using population-level data for dense SNPs in ten selected genomic regions on ten different chromosomes.

## Materials and Methods

### Samples and genotypes

**Fig 1:**
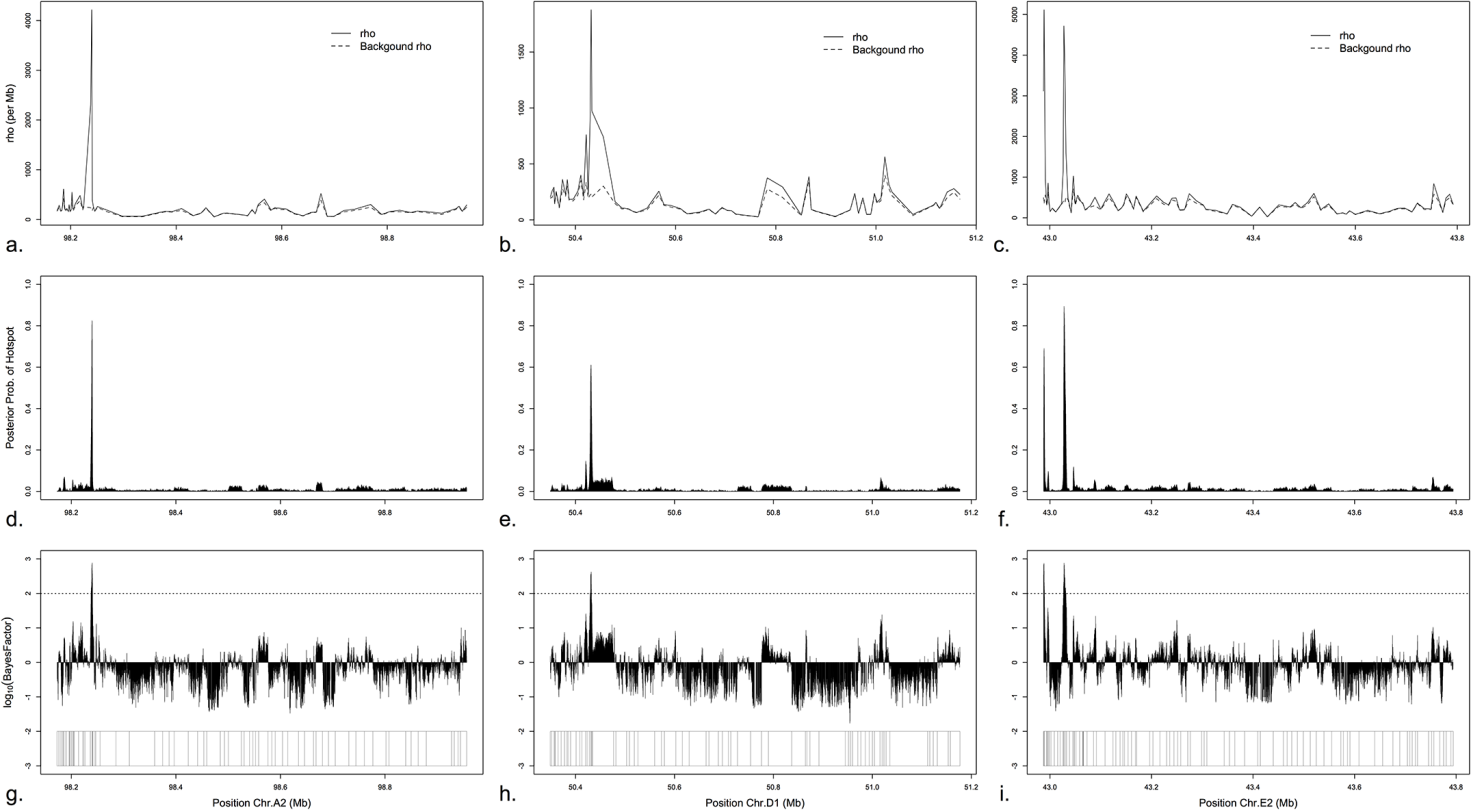
Recombination overview of chromosomes A2, D1, and E2 regions. (a-c) Posterior recombination rates (population size scaled) across chromosomes A2, D1, and E2 regions, respectively. Solid line shows the whole recombination rates, while the dashed line shows the background recombination rates. (d-f) Posterior probability of hotspots along chromosomes A2, D1, and E2 regions, respectively. (g-i) Bayes factor of hotspots for chromosomes A2, D1, and E2 regions, respectively. Horizontal dotted line corresponds to Bayes factor of 100 in a log_10_ scale. Position and distribution of SNPs included in the recombination analysis are at the bottom.

SNP genotype data of twenty-two feral cats from China were obtained as previously described in [23]. The data were generated using a custom Illumina GoldenGate array that represent ten different cat chromosomal regions (A1, A2, B3, C2, D1, D2, D4, E2, F2, X) and were composed of 1536 markers nearly equally distributed across the ten regions. Markers’ positions were updated to their location in the 6.2 cat genome assembly (http://genome.ucsc.edu/). To ensure successful inference, three criteria were used to filter the SNPs: (i) SNPs mapped to a single chromosomal location on the most recent genome assembly of cat with 100% sequence match, (ii) SNPs exhibiting a genotype call success rate of ≥ 80%, and (iii) SNPs possessing a minor allele frequency of ≥ 0.1. The final dataset, included in this study, is composed of 701 markers distributed over the ten regions (S1 Table). The genomic locations of the regions and related summaries are shown in Table 1 (see also Fig 1 and S2 Fig).

**Table 1:** Summary of the chromosomal regions analyzed for recombination in cats.

Chr.	No. SNPs	Position (bp) ^1^		Region size (bp)	Mean inter-SNP distance	Mean Rho (*ρ*/Mb)	Number bins with BF ^2^		
		Start	End				< 100	≥ 100	Total
A1	53	187 299 691	188 082 901	783 210	15 061	127.68	3915	0	3915
A2	73	98 172 535	98 956 464	783 929	10 890	186.51	3904	15	3919
B3	88	126 081 886	126 859 181	777 295	8 934	202.67	3886	0	3886
C2	53	4 360 303	5 085 917	725 614	13 950	291.85	3628	0	3628
D1	70	50 348 845	51 177 183	828 338	12 000	176.72	4131	10	4141
D2	86	59 655 061	60 470 312	815 251	9 591	192.93	4074	0	4074
D4	73	45 514 026	46 304 562	790 536	10 980	259.47	3951	0	3951
E2	90	42 987 235	43 794 319	807 084	9 068	309.48	4002	32	4034
F2	78	10 965 562	12 451 350	1 485 788	19 300	185.95	7427	0	7427
X	37	100 018 322	100 740 914	722 592	20 070	77.45	3613	0	3613
**Mean**	**70**	**-**	**-**	**851 964**	**12 984**	**201**	**4253**	**6**	**4259**
**Total**	**701**	**-**	**-**	**8 519 637**	**-**	**-**	**42531**	**57**	**42588**

^1^ Start and end positions are based on the 2011 cat genome assembly (ICGSC Felis_catus 6.2)

^2^ BF: Bayes factor.

Three SNPs residing in a recombination hotspot on chromosome E2 region (see results) were chosen for genotype validation via sequencing. Primers were designed to flank the SNPs (S2 Table) and PCR was performed using DNA Engine Gradient Cycler (MJ Research, GMI, Ramsey, MN) using the following conditions. For each reaction, 2μl of DNA was used in a 1.5mM magnesium concentration with 1μM primers in a total reaction volume of 20μl. The annealing temperature of all primers was 62°C. The PCR protocol was as follows: initial denaturation at 94°C for 5 min followed by 40 cycles of 94°Cx45 sec, 62°Cx20 sec, 72°Cx30 sec, and a final extension at 72°C for 20 min. The PCR products were purified with ExoSap (USB, Cleveland, OH) per the manufacture’s recommendations and directly sequenced using the BigDye terminator Sequencing Kit v3.1 (Applied Biosystems, Foster City, CA) as previously implemented [24]. Sequences were verified and aligned using the software sequencer version 4.10 (Gene Codes Corp., Ann Arbor, MI).

### Recombination inference

The program *inferRho* uses the coalescent with recombination model [25–27] in a full Bayesian framework to infer recombination rates and hot spots along the chromosome regions [28,29]. The evolutionary relationship of the SNPs is represented by the ancestral recombination graph (ARG), which is an unobserved random variable that is integrated over using Markov Chain Monte Carlo (MCMC). In the variable recombination rate model, the population-scaled recombination rate is *ρ*_*i*_ = 4*N*_*e*_*c*_*i*_, where *N*_*e*_ is the effective population size, *c*_*i*_ is the recombination rate per generation in cM/Mb between marker *i* and *i* +1 (*i* = 1,…, *k* − 1, and *k* is the total number of SNPs). The total recombination rate (*c*_*i*_) consists of the background crossing-over rate and recombination hotspots (arising according to a Markov process) [28,29]. As genetic data (SNPs) from a population sample of unrelated individuals only provide information about evolutionary distance, the parameters in coalescent estimators are typically scaled by the effective population size (i.e. *θ* = 4*N*_*e*_*μ*, where *μ* is the substitution rate per site per generation; and *ρ* = 4*N*_*e*_*c*). To convert *ρ* into standard unit (cM/Mb), we need to divide it by the ploidy factor and effective population size (4*N*_*e*_ for the nuclear genome of cats), which typically comes from other sources of information.

To make the data analysis computationally tractable, each chromosomal region was divided into blocks, with 20 SNPs per block (e.g., chromosome A1 region with 53 SNPs was divided into 3 blocks, 20 SNPs in the first and second block and 13 SNPs in the third block). Blocks from each chromosomal region are assumed to share the same population size parameter (*θ*) but have an independent ARG for each block. Preliminary runs were executed to determine the appropriate length and thinning interval of the MCMC chain for each chromosomal dataset. The last half of the MCMC samples (1000 samples) was used to estimate the recombination rates (*ρ*_*i*_) and to plot them with respect to the SNP marker positions. The mean population-scaled recombination rate per site for each chromosomal region was calculated using the following equation:

$$\bar{\rho }=\sum_{i=1}^{k-1}\rho_{i}d_{i}/\sum_{i=1}^{k-1}d_{i},$$

in which *d*_*i*_ is the distance between marker *i* and *i*+1, and *k* is the total number of markers in the region. The numerator is total population-scaled recombination rate of the region, while the denominator is the length of this region in Mb.

### Designation of recombination hot spots

We calculated the Bayes factors [30] to locate the positions of hotspots and represent relative odds of a hotspot being present. Each chromosomal region was divided into bins (200 bp per bin) for analysis of posterior samples, which contain the start and end positions of the hotspots, to estimate the probability of having hotspot (*p*_*j*_) for each bin. The corresponding probability of hotspot for each bin (*q*_*j*_) from the prior was obtained by running the program without data (e.g., constant likelihood) but maintaining the same sample size and marker positions. The Bayes factor (*BF*) is defined as the ratio of the posterior and prior odds:

$$BF=\frac{p_{j}/\left(1-p_{j}\right)}{q_{j}/\left(1-q_{j}\right)}.$$

The odds ratio measures the proportional change in odds favoring a hotspot in the region (versus the prior odds) that results from including the data (SNPs). The total number of bins depends on the length of the region; thus the range of *j* is variable among the ten chromosome regions. Recombination hotspots are defined to have at least two consecutive bins with *BF* ≥ 100 whereas regions of *BF* < 100 are denoted as “neutral” bins awaiting further investigation (cf. section 3.2 in [30]).

### GC content and genomic elements analyses

The GC content was calculated for the sequences of each bin using the function *CG*.*content* of package *APE* in R [31].

Variation and repeat elements within each of the ten chromosomal regions were downloaded from UCSC genome browser using *RepeatMasker* for the v6.2 cat genome assembly. Elements were analyzed separately for bins with *BF* < 100 and *BF* ≥ 100 (hotspot bins). The elements within neutral bins (*BF* < 100) were to provide the general overview of the elements within each chromosomal region.

## Results

### Samples and genotypes

The dataset is composed of 701 SNPs on ten different cat chromosomal regions with an average of 70 SNPs/region. The chromosome E2 region harbors the highest number of SNPs (*n* = 90) while chromosome X region has the lowest (*n* = 37). SNPs are distributed across the regions with an average distance between SNPs of 12 Kb and a range of 145 bp– 651 Kb (Table 1). Four SNPs were selected for genotype verification using direct sequencing. The sequencing results were concordant to the genotypes obtained previously using the genotyping platform.

### Recombination rates and hotspots

The ten regions on chromosomes: A1, A2, B3, C2, D1, D2, D4, E2, F2, and X, were analyzed using *inferRho*. The lengths of the MCMC chains were determined by the trace plot and effective sample size (ESS) of the parameters as: 600 000 iterations for A1, C2; 800 000 iterations for A2, D1, D4, F2; 1 000 000 iterations for B3, D2, E2; and 500 000 iterations for X. Two thousand posterior samples for each chromosomal dataset were obtained after thinning, while the first half was discarded as burn-in. Using a parallel computing approach, each run could be accomplished within one to two weeks on a cluster with 2 Opteron 270 (2.0 GHz) processors per node.

Population recombination rate (*ρ*) is plotted for each region (Fig 1a–1c and S1a Fig). The mean recombination rate (*ρ*) across all regions is 200 per Mb. The E2 region exhibits the highest mean rate (309 /Mb) whereas X region has the lowest (77 /Mb) (Table 1). The difference between the background recombination rate and whole recombination rate is indicative of recombination spots. This difference is most noticeable in regions of chromosomes A2, D1, and E2 (Fig 1a–1c).

The posterior probability of hotspot was calculated for bins of size 200 bp in each region (Fig 1d–1f and S1b Fig). Chromosomes A2, D1, and E2 show distinctly high posterior probabilities (> 0.6) in four localized areas (Fig 1d–1f). The posterior probabilities for the other seven chromosome regions (A1, B3, C2, D2, D4, F2, and X) are less than 0.2, indicating little support for elevation of recombination rate in these areas.

The Bayes factors (Fig 1g–1i and S1c Fig) are consistent in pattern with the posterior probabilities (Fig 1d–1f and S1b Fig) and recombination rates (Fig 1a–1c and S1a Fig). Approximately 99% (*n* = 42,531) of bins were classified as “neutral” (*BF* < 100) across all regions examined. The hot spots were found in only three chromosomal regions (A2, D1, and E2) and represented ~ 0.13% (*n* = 57) of all bins studied. Summaries of the numbers and distribution of bins within each chromosomal region are provided in Table 1. The hotspots had size of 3 Kb in A2, 1.8 Kb in D1, and 1.8 Kb for the first and 4.6 Kb for the second in E2. The distance between the two hotspots on E2 was ~37.4 Kb (Table 2).

**Table 2:** Summary of recombination “hotspots” in cats selected regions.

Chr.	Position (bp)		Size (Kb)	# bins	Mean			Elements					
	Start	End			PP	BF	GC	LINE	SINE	LC	LTR	SR	DNA
A2	98 237 700	98 240 700	3	15	0.54	339.85	0.35	L1MEg	SINEC-Fc2 MIR MIRb	AT-rich	-	-	MER46C
D1	50 430 400	50 432 200	1.8	9	0.56	271.90	0.38	L2a	SINEC-Fc2 MIRb (2)	-	MLT1J	(TA)n	-
E2	42 987 500	42 989 300	1.8	9	0.54	418.27	0.40	L2a	MIR3	-	-	-	-
E2	43 026 700	43 031 300	4.6	23	0.65	293.30	0.42	L2b (2) L2a	SINEC-Fc2 MIR	-	-	(GA)n (TG)n	MER20
		**Mean**	**2.80**	**14**	**0.57**	**330.83**	**0.39**						

PP: mean posterior probability of hotspot,

BF: mean Bayes factor,

GC: mean GC content within interval,

Elements: variation and repeat elements within interval (start and end positions)

(LINE: long interspersed element,

SINE: short interspersed element,

LC: low complexity,

LTR: long terminal repeat,

SR: simple repeat,

DNA: MER—Medium reiterated repeats).

### GC content analysis

The log_10_ of the Bayes factor was plotted as a function of the GC content in S2 Fig. Pearson’s correlation test revealed a positive correlation between GC and log_10_(Bayes factor) (cor = 0.077, *p* < 0.0001) but was not suggestive of strong correlation. Moreover, no significant differences in the mean GC content of each class of bins were observed (t-test, *p* = 0.05) (S2 Fig). The mean GC contents of the hotspots are shown in Table 2.

### Repeat elements analysis

**Fig 2:**
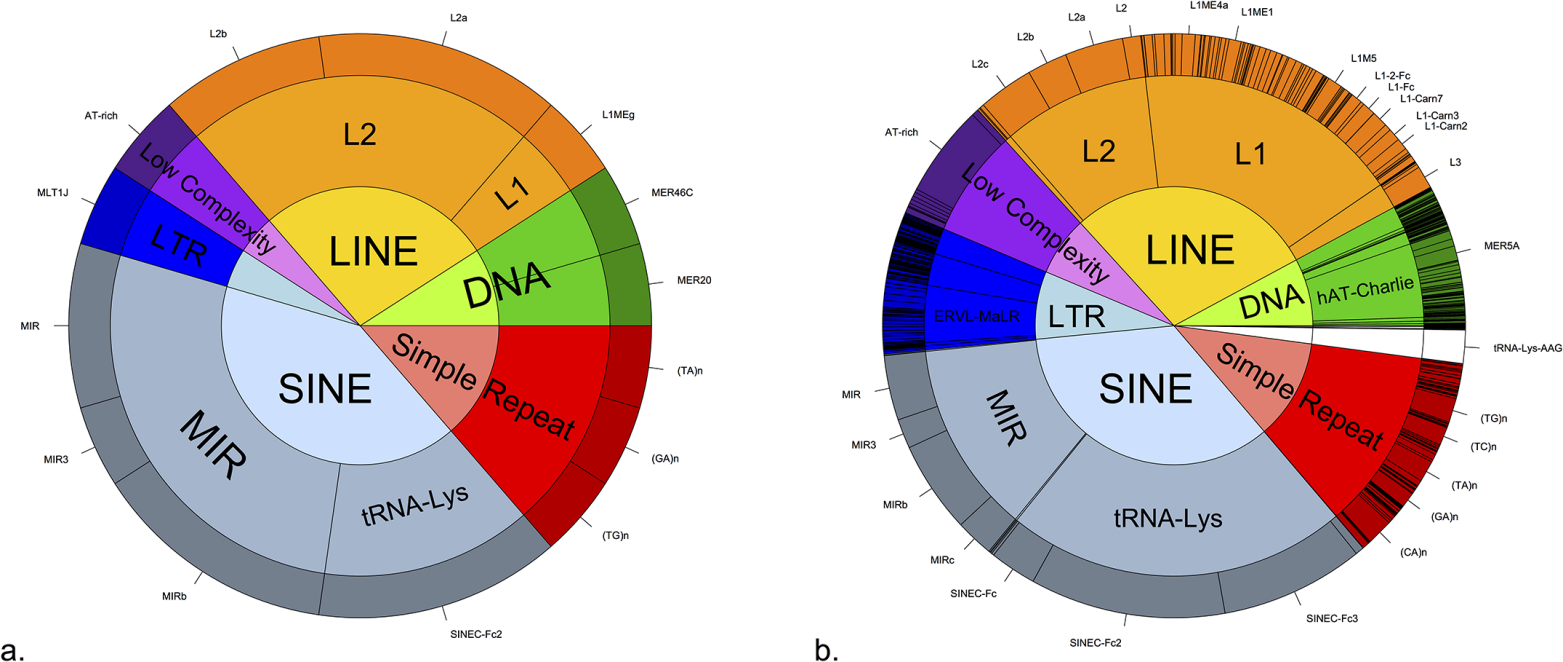
A qualitative overview of variation and repeat elements in the cat chromosomal regions studied. a) Variation and repeat element (*n* = 22) within four hotspots. All elements within the hotspots are shown at the tips of the outer circle. b) Variation and repeat element (*n* = 16,798) within “neutral” bins. Elements present more than a hundred times within the “neutral” bins are shown at the tips of the outer circle. The inner circle represents the elements’ classes, middle circle represents elements’ families, and outer circle represent the individual elements. Note: the Fig is intended a general description rather than a quantitative comparison.

The four hotspot regions contained 22 repeat and variation elements (Table 2, Fig 2a). SINE elements constitute the highest proportion (40%) of the elements present in the hotspots followed by LINE elements (27%). Within LINE elements, L2 elements were present in three of the four hotspot regions. MIR family elements were present in all hotspot regions and tRNA-Lys family elements are present in three of the four elements. Low complexity, long terminal repeats, simple repeats, and DNA elements were inconsistently present across the hotspot regions.

Variation and repeat elements within neutral regions where investigated to get a picture of the general distribution of repeat elements (Fig 2b). In neutral regions, the SINE elements represent the highest proportion of elements, 34%. The tRNA-Lys SINE elements constitute 22% and MIR SINE elements represent 12%. The second highest repeat elements was the LINE elements, 29%, where L1 elements represent ~17% and L2 elements represent 9.5% of all elements in the neutral regions.

## Discussion

Advances in population genetic theory and technology supports the estimation of recombination rates directly from genotype data on population samples, overcoming the limitations of sperm typing or using large extended families [32]. The strategy of the population genetics based approach is to use information on the number of recombination events that have occurred in the history of the population, which can be detected by modeling the patterns of genetic variation expected to be present in randomly selected individuals.

The model accounts for coalescent and recombination events in an Ancestral Recombination Graph (ARG). Markers will have coalescent trees that are likely to vary across the genome. In theory, all markers in a chromosome are correlated by an ARG. However, the size of the ARG may grow much faster than linear with the increasing number of SNPs, making it computational intractable to simultaneously analyze all markers in an analysis. In practice, the data are usually partitioned into blocks. Blocks from the same chromosomal region are assumed to share the same population size parameter (*θ*) but have an independent ARG for each block. For fast computation, some methods use an approximate likelihood instead of the full likelihood calculation, for example, the composite-likelihood method implemented in *LDhat* [33] and PAC-likelihood method implemented in *PHASE* [34]. Approximate-likelihood methods may be feasible to apply to large genomic regions, but may lack power to detect a moderate or low rate of recombination. Full-likelihood methods, such as implemented in *inferRho*, use all the information in the data and should therefore provide more accurate estimates [28,29].

The analysis of recombination hotspots in cats, presented here, constitutes the first application of the program *inferRho* to non-human data and the first analysis of fine-scale recombination rates in cats. The population recombination rate was found to be variable between the regions analyzed and, as expected, the mean recombination rate of X chromosome regions was lower than that of any autosomal regions. This variation in recombination rates and the notable reduced rate outside of the pseudoautosomal region of the X chromosome are in agreement with observations of recombination in human [4] and dog [7]. The latter result is expected due to that fact that recombination outside of the pseudoautosomal region occurs only in females.

Four decisive hot spots were identified on three chromosomes: A2, D1 and E2. The localities of the hotspots are in agreement with the localities of increased posterior probabilities and the general topography of recombination rates as expected. Acknowledging the limitation posed by the total size of the regions analyzed (total ~ 8.5 Mb) compared to the size of the genome and the lack of power to perform correlation and element enrichment analyses, the following observations have been made: (i) the cat hotspots, identified in this study, show no distinct positive correlation with GC content. (ii) The four hotspots contain at least one L2 LINE element and MIR and tRNA-Lys SINE elements. (iii) The similarity of the repeat elements in cat hotspots compared to other mammals might suggest similar recombination mechanisms.

This study represents a glimpse of recombination hotspots in cats and only an initial step toward understanding recombination in cats. The markers are sparsely sampled in some of the genomic regions, which may reduce power for *inferRho* to detect a large number of hotspots. As cat resources develop, genome-wide analyses could be performed allowing more definitive conclusions to be reached. Nonetheless, our preliminary description of the recombination landscape, and our finding that hotspots are present, helps shed light on the mechanism of recombination in cats compared to other species, furthering our understanding of the patterns of variation generated by recombination in cats, and potentially leading to better implementation of efficient disease mapping strategies in cats.

## Supporting Information

S1 Fig Recombination overview of seven chromosomal regions. (a) Estimated recombination rates (population size scaled crossing-over rate) along each region. Solid line shows the estimated recombination rates between markers, while the dashed line shows the background recombination rates. (b) Posterior probability of hotspots across each region. (c) Bayes factor of hotspots along each region. Horizontal dashed line corresponds to Bayes factor of 10 in a log_10_ scale.
(JPG)

S2 Fig Analysis of GC content of recombination spots in cats. (a) The GC contents (x-axis, 200 bp each bin) against the log_10_ of Bayes factors (y-axis). Gray circles represent neutral bins with *BF* < 100, and black circles represent hotspot bins with *BF* ≥ 100. (b) Boxplot of the GC content in the two classes of bins listed in (a).
(JPG)

S1 Table SNP genotype data used to infer recombination hotspots in selected regions of the cat genome. (XLSX)

S2 Table Primers of selected SNPs located in chromosome E2 hotspot. (DOC)
