# Supplementary figures and images for: A Glance at Recombination Hotspots in the Domestic Cat
Source: PLoS One. 2016 Feb 9;11(2):e0148710. doi: 10.1371/journal.pone.0148710 (PMC4747544; doi:10.1371/journal.pone.0148710)

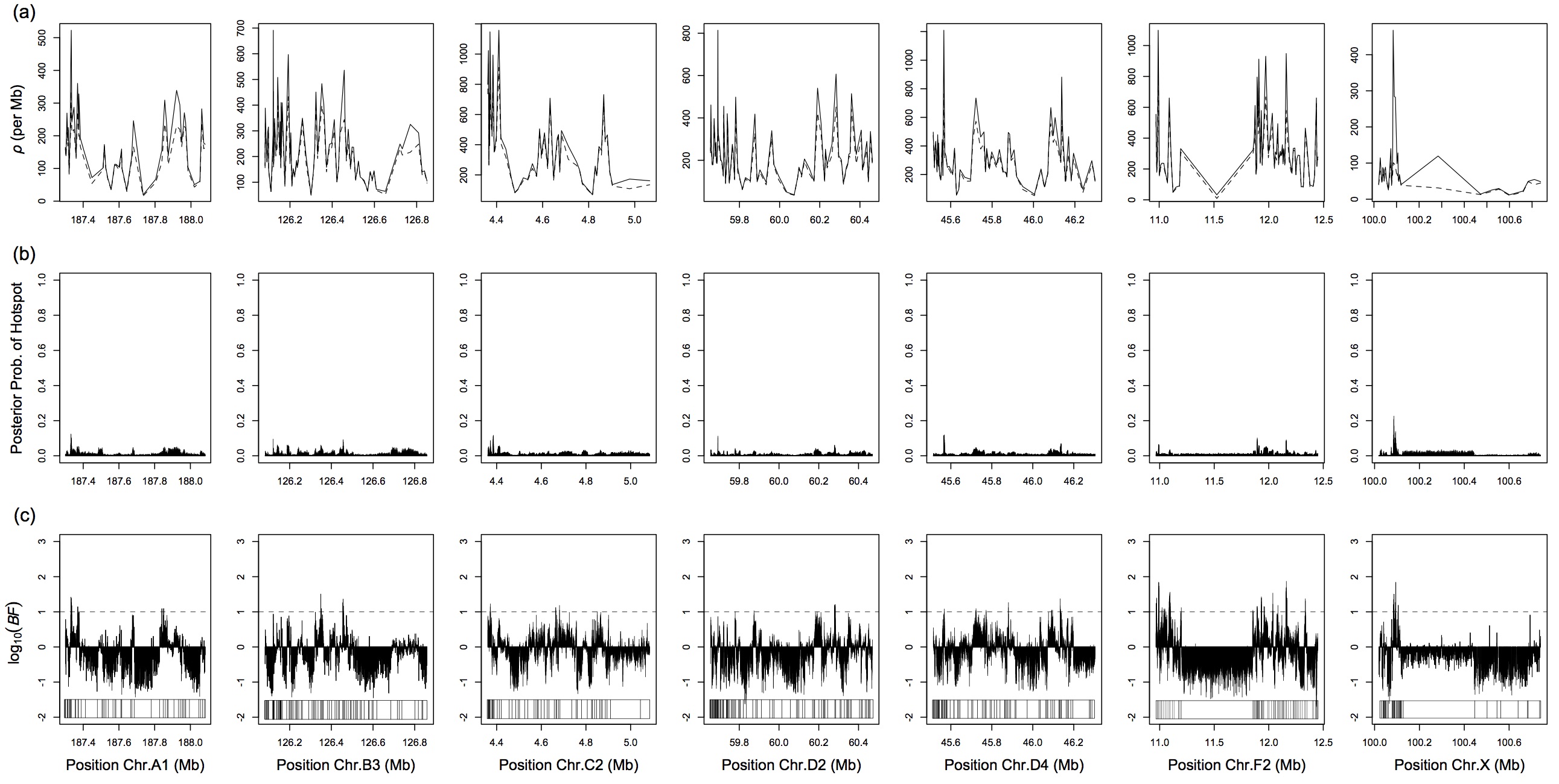

Supplement: S1 Fig — (a) Estimated recombination rates (population size scaled crossing-over rate) along each region. Solid line shows the estimated recombination rates between markers, while the dashed line shows the background recombination rates. (b) Posterior probability of hotspots across each region. (c) Bayes factor of hotspots along each region. Horizontal dashed line corresponds to Bayes factor of 10 in a log10 scale. (JPG) [file pone.0148710.s001.jpg]

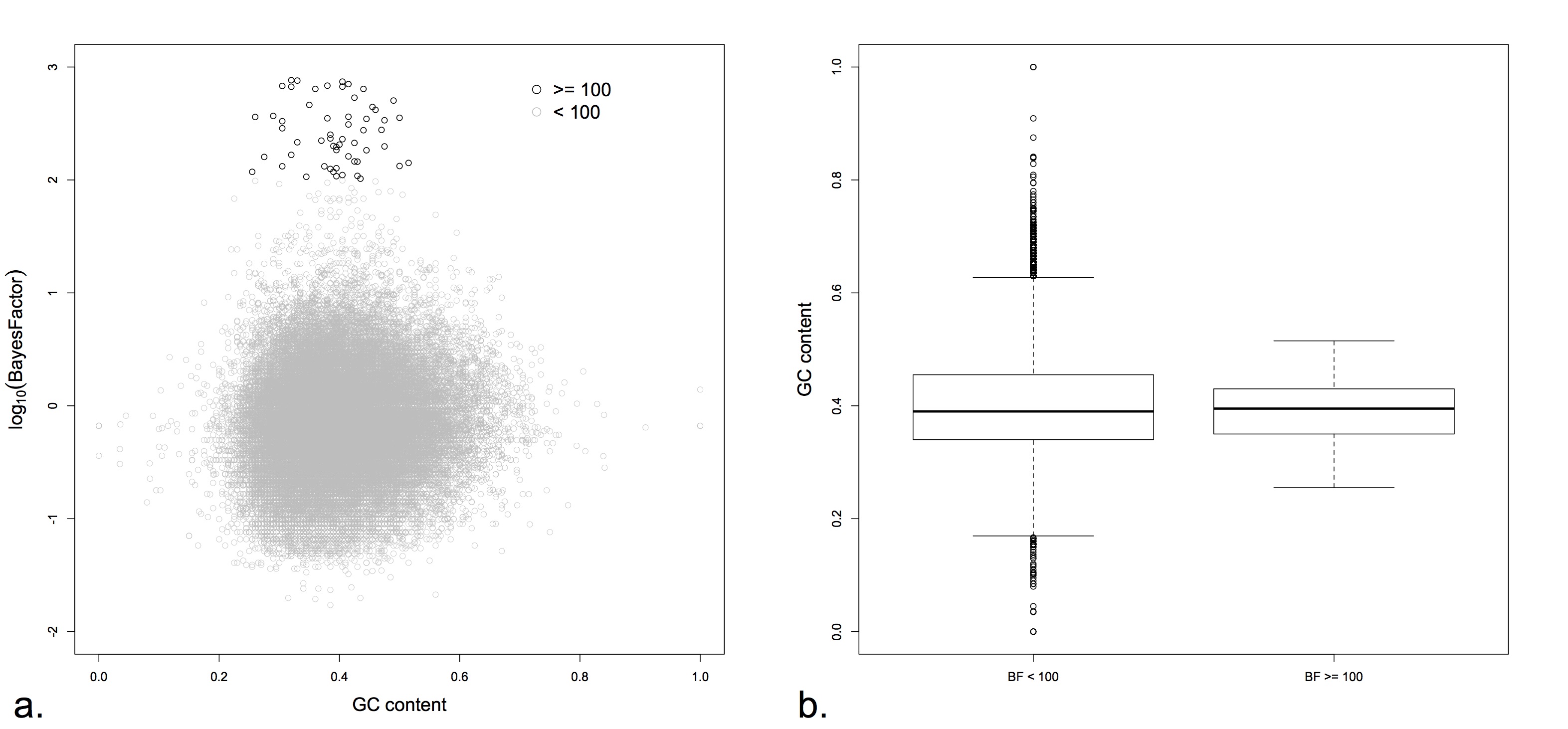

Supplement: S2 Fig — (a) The GC contents (x-axis, 200 bp each bin) against the log10 of Bayes factors (y-axis). Gray circles represent neutral bins with BF < 100, and black circles represent hotspot bins with BF ≥ 100. (b) Boxplot of the GC content in the two classes of bins listed in (a). (JPG) [file pone.0148710.s002.jpg]
