# Supplementary material for: A Glance at Recombination Hotspots in the Domestic Cat
Source: PLoS One. 2016 Feb 9;11(2):e0148710. doi: 10.1371/journal.pone.0148710 (PMC4747544; doi:10.1371/journal.pone.0148710)
Supplement: S2 Table — (DOC) [file pone.0148710.s004.doc]

**Supplementary Table 2:** Primers of selected SNPs located in chromosome E2 hotspot.

| **Chr.** | **SNP ID** | **Position (bp)** | **Primers** | |
| --- | --- | --- | --- | --- |
| E2 | chrE2.51564858 | 43025566 | **F** | CTGGCAAACACTTATTGTGAAT |
|  |  |  | **R** | CAGAATGGTGCATCAGCTGTATGT |
| E2 | chrE2.51566221 | 43026930 | **F** | ATAGGGGATATTTAAAATGCCTTCTAAT |
|  |  |  | **R** | ATGAAACATGTCAGTGGCTTGGT |
| E2 | chrE2.51567534 | 43028243 | **F** | AATTTCAGGGATTTGTAGAAGATTGTT |
|  |  |  | **R** | TGCTAAGGTTGTCACCTGTTTTCA |
